# Hospitalization burden and epidemiology of the COVID-19 pandemic in Spain (2020–2021)

**DOI:** 10.1186/s12879-023-08454-y

**Published:** 2023-07-18

**Authors:** Rafael Garcia-Carretero, Oscar Vazquez-Gomez, Ruth Gil-Prieto, Angel Gil-de-Miguel

**Affiliations:** 1 Department of Internal Medicine, Mostoles University Hospital, Rey Juan Carlos University, Madrid, Spain; 2 Department of Preventive Medicine and Public Health, Rey Juan Carlos University, Madrid, Spain

**Keywords:** COVID-19, Health care, SARS-CoV-2

## Abstract

**Background:**

Spain had some of Europe’s highest incidence and mortality rates for coronavirus disease 2019 (COVID-19). Here we describe the epidemiology and trends in hospitalizations, the number of critical patients, and deaths in Spain in 2020 and 2021.

**Methods:**

We performed a descriptive, retrospective, nationwide study using an administrative database, the Minimum Basic Data Set at Hospitalization, which includes 95–97% of discharge reports for patients hospitalized in Spain in 2020 and 2021. We analyzed the number of hospitalizations, admissions to intensive care units, and deaths and their geographic distribution across regions of Spain.

**Results:**

As of December 31, 2021, a total of 498,789 patients (1.04% of the entire Spanish population) had needed hospitalization. At least six waves of illness were identified. Men were more prone to hospitalization than women. The median age was 66. A total of 54,340 patients (10.9% of all hospitalizations) had been admitted to the intensive care unit. We identified 71,437 deaths (mortality rate of 14.3% among hospitalized patients). We also observed important differences among regions, with Madrid being the epicenter of hospitalizations and mortality.

**Conclusions:**

We analyzed Spain’s response to COVID-19 and describe here its experiences during the pandemic in terms of hospitalizations, critical illness, and deaths. This research highlights changes over several months and waves and the importance of factors such as vaccination, the predominant variant of the virus, and public health interventions in the rise and fall of the outbreaks.

## Introduction

A novel coronavirus disease was first described in 2019 and named 2019-nCoV. Its denomination was later replaced by severe acute respiratory syndrome coronavirus-2 (SARS-CoV-2). This virus is the causative agent of coronavirus disease 2019 (COVID-19) [1]. This disease can range from mild symptoms to severe viral pneumonia with acute respiratory failure and distress syndrome, also known as severe acute respiratory syndrome [1, 2]. Although the World Health Organization reported cases of pneumonia in Wuhan City, Hubei Province, China, as early as 2019, the outbreak was finally declared a public health emergency in January 2020 and a pandemic in March 2020 [3].

Outbreaks of COVID-19 usually spread in regular patterns, but waves of the disease vary widely among different countries and regions within a single country. During the first weeks of the pandemic mortality rates in Spain and Italy rose to 15%, in contrast to countries such as Canada and Germany, whose mortality rates were less than 5% in the first wave [4–8]. The impact and the distribution of these waves depend on several factors, such as public health measures, interventions, lockdowns, and vaccination policies [9, 10]. SARS-CoV-2 variants can also have a great effect on mortality, because they can increase the transmissibility of the virus and clinical severity of the disease or decrease the effectiveness of public health interventions [11, 12].

Demographic data on the first weeks of the pandemic in Spain were reported early and provided a global overview of COVID-19 [13]. Spain reported its first confirmed case in January 2020. The epidemic escalated quickly and spread across all regions of the country. A state of emergency was finally declared in March 2020. Spain reported not only one of the highest incidence rates in Europe but also one of the highest mortality rates, accounting for 172,541 confirmed cases and 18,056 deaths by the end of April 2020 [14], corresponding to the first wave of the pandemic.

Describing and analyzing trends in the pandemic at a national level can help researchers and public health authorities obtain new insights into the pandemic. We collected all available data from the first 2 years of the pandemic to explore trends and the impact of the pandemic in Spain. Our aim was to analyze the behavior of the pandemic throughout its first 2 years at a national level. Here we describe the distribution of hospitalizations and admissions to intensive care units (ICUs) and the mortality rate by region in Spain.

## Methods

### Study design and data collection

We performed a nationwide, population-based, epidemiological study of all hospitalizations due to COVID-19 from February 2020 to December 2021. We used the Minimum Basic Data Set at Hospitalization (MBDS-H), which is an administrative database based on hospital discharge reports. The registry is mandatory for all public and private hospitals and so covers 95–97% of hospitalizations in Spain. This database is built from hospital discharge reports [15]. The MBDS-H includes not only demographic information, such as age, sex, and the province where hospitalization occurred, but also the date of admission to the hospital, the date of discharge, mortality, and ICU admission. There are two variables of interest: primary and secondary diagnosis. Data are available on reasonable request from the statistical portal of the Spanish National Health System [16] and are provided without personal data that could be used to identify patients to ensure their privacy.

Diagnoses in the data set are coded according to the International Classification of Diseases, 10th Revision, Clinical Modification (ICD-10-CM) [17]. We used codes U07.1 and B97.29 to extract the data. Because the data are based on hospital admissions, patients who either were discharged from the emergency room or were seen in outpatient settings are excluded. We included data from individuals whose primary or secondary diagnoses included codes U07.1 and/or B97.29 (both referring to COVID-19, according to the ICD-10-CM) in 2020 or 2021. As of this writing, data from 2022 are not yet available.

The Spanish National Health System updated the national coding recommendations, aligning them with the ICD-10-CM Guidelines for coding and reporting. Specifically, the code U07.1 COVID-19 has been recommended by the ICD-10-CM for encoding COVID-19 infections. Consequently, all records from April 1st, 2020, onwards should be encoded using this new code. In Spain, considering the overwhelmed state of Spanish hospitals, it was decided, after assessment by the Encoding Technical Unit (Unidad Técnica de Codificación), to implement the use of this new code starting from July 2020. This ensured the consistent use of data across the country by professional coders. This encoding change was taken into account during the preprocessing of our database.

During the initial months of the pandemic, the coding procedure involved considering the primary diagnosis along with the first secondary diagnosis. For instance, pneumonia due to COVID-19 was identified using codes J12.89 (other viral pneumonia) and B97.29 (other coronavirus as the cause of diseases classified elsewhere). However, since July 2020, the code U07.1 COVID-19 should be used only when a positive test result for SARS-CoV-2 has been confirmed. It should not be used if the testing is suspected, likely, or inconclusive. According to the recommendations, U07.1 should be encoded as the primary diagnosis, while the secondary diagnosis should be used to describe the clinical manifestations. For example, “U07.1 COVID-19 + J12.89” should be used to describe pneumonia due to COVID-19. Our database underwent rigorous quality control at the Encoding Technical Unit (Spanish Ministry of Health) before being provided to investigators. We have taken all these aspects into consideration in order to conduct our analyses effectively.

### Statistical analyses

We designed our research to be a descriptive, retrospective study. In order to represent the data from the data set, various statistical methods were employed. Firstly, absolute values were extracted to gain a clear understanding of the actual numbers involved, such as the total hospital admissions and ICU admissions. This allowed for a comprehensive overview of the data set. Additionally, percentages were calculated to provide a relative perspective, enabling comparisons between different variables. These percentages were computed for variables such as age groups, gender distribution, and geographic regions, shedding light on the proportional representation within each category. Although no correlational analysis was conducted, this descriptive statistical approach successfully facilitated the presentation and interpretation of the data set.

We performed the Anderson-Darling normality test to check the distribution of continuous variables, such as age, and report data as means and standard deviations or medians and interquartile ranges. Categorical variables are reported as absolute values and percentages. Differences between the years were assessed with the chi-square test for categorical variables and the Mann-Whitney U test for continuous variables. All statistical analyses were performed in R 4.2.2 (2022-10-31) on a GNU/Linux computer. For statistical significance, we used a p value of 0.05.

## Results

### Global overview

**Fig. 1:**
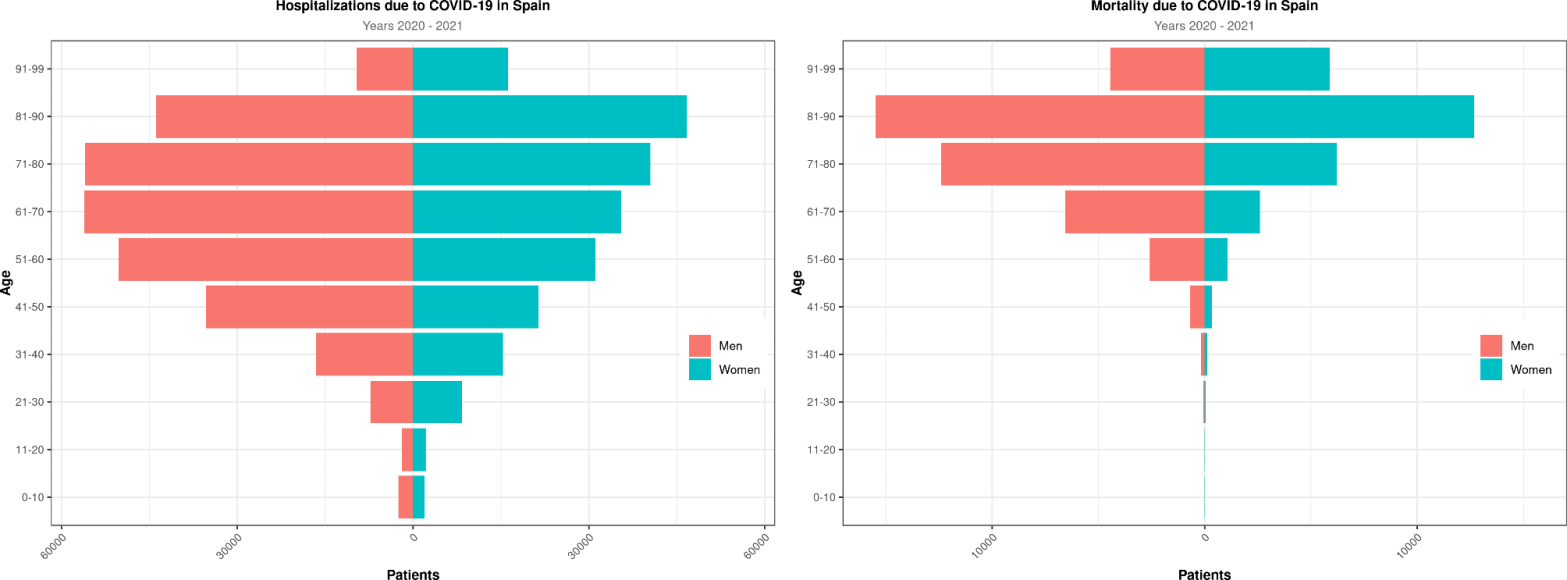
Population pyramids for hospital admissions and mortality

A total of 498,789 patients (1.04% of the Spanish population) were admitted to hospitals with a diagnosis of COVID-19 in the first 2 years of the pandemic. Table 1 shows the results by year. Men were more prone to hospitalization than women in this period. The median age was 66, but we found that patients were slightly older in 2020 than in 2021 (68 vs. 65, respectively). Hospital stay was longer in 2020 (median, 11.2 days) than in 2021 (median, 8 days). A total of 54,340 patients, or 10.9% of all hospitalizations, were admitted to the ICU. We also observed a global mortality rate of 14.3% among hospitalized patients, with 71,437 total deaths. The mortality rate was higher in 2020 (16.1%) than in 2021 (12.5%). Table 2 shows ICU admissions (as a surrogate of severity) and in-hospital mortality by different age groups. Figure 1 shows population pyramids for both hospital admissions and mortality. Men were more represented than women among both admissions and mortalities in almost all age ranges, except among patients older than 90 years old. Mortality was most frequent in patients ages 70 to 90 years old.

**Table 1:** Overview of the main characteristics of hospitalized patients

	Total	2020	2021	p value
**Patients**	498,789	252,176	246,613	NA
**Sex (male, %)**	56.1	55.6	56.5	< 0.001
**Age (years)**	66 (28)	68 (27)	65 (28)	< 0.001
**Hospital stay (days)**	8 (9)	11.2 (9)	8 (9)	< 0.001
**ICU (patients)**	54,354	22,949	31,405	< 0.001
**ICU (%)**	10.9	9.1	12.7	< 0.001
**ICU stay (days)**	10.0 (21)	10 (16.7)	11 (23.5)	< 0.001
**Deaths**	71,437	40,512	30,925	< 0.001
**Mortality rate (%)**	14.3	16.1	12.5	< 0.001

NA: not applicable. ICU: intensive care unit. Data are expressed as percentages for categorical variables and as medians (interquartile ranges) for continuous variables

**Table 2:** Admissions, ICU admissions and mortality by age group

	2020					2021				
Age groups	Total Admissions	ICU Admissions	ICU (%)	Deaths	Mortality rate (%)	Total Admissions	ICU Admissions	ICU (%)	Deaths	Mortality rate (%)
**< 14**	1,978	177	8.9	6	0.3	3,475	205	5.9	14	0.4
**15–44**	29,618	2,279	7.7	343	1.2	41,106	4,400	10.7	355	0.9
**45–64**	77,489	9,711	12.5	3,764	4.9	78,126	13,221	16.9	3,631	4.6
**65–74**	47,373	7,151	15.1	6,688	14.1	45,818	9,287	20.3	5,784	12.6
**> 74**	95,821	3,659	3.8	29,743	31	78,098	4,292	5.5	21,147	27.1

ICU: intensive care unit. Data are expressed as percentages and absolute values

### Evolution of the pandemic

**Fig. 2:**
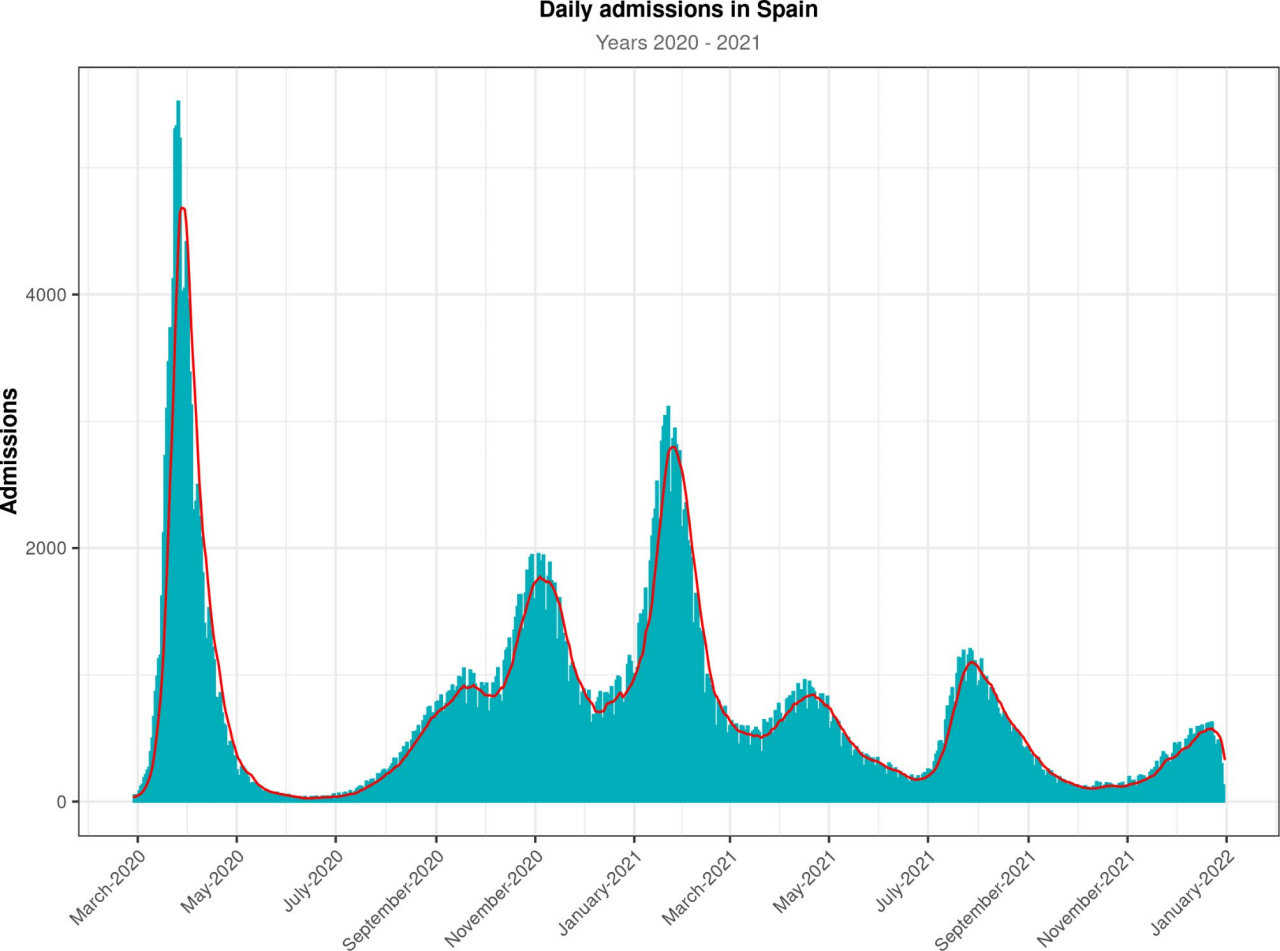
Time series showing the evolution of COVID-19-related hospitalizations in Spain in 2020 and 2021. The red line denotes the 7-day moving average, whereas the gray bars denote absolute numbers of hospital admissions

In the period observed, we identified at least six waves of hospitalizations (Fig. 2). It is worth noting that one third of all hospital admissions occurred within the first 12 weeks of the pandemic.

### Distribution of the pandemic by region

The geographic distribution of the pandemic in the different regions of Spain can be seen in Table 3, which shows geographic variability among regions. During the first year of the pandemic, most hospitalizations were in Madrid (70,105 admissions) and Catalunya (51,237 admissions). Overall, in all regions, men were more likely than women to be hospitalized in both 2020 and 2021. The median age was above 60 in all regions (68 globally) in 2020, but in 2021 patients tended to be younger, with a global median age of 65 (in only one region above 70). Overall, sex, age, and hospital stay were consistent among regions of Spain and between years in a given region.

**Fig. 3:**
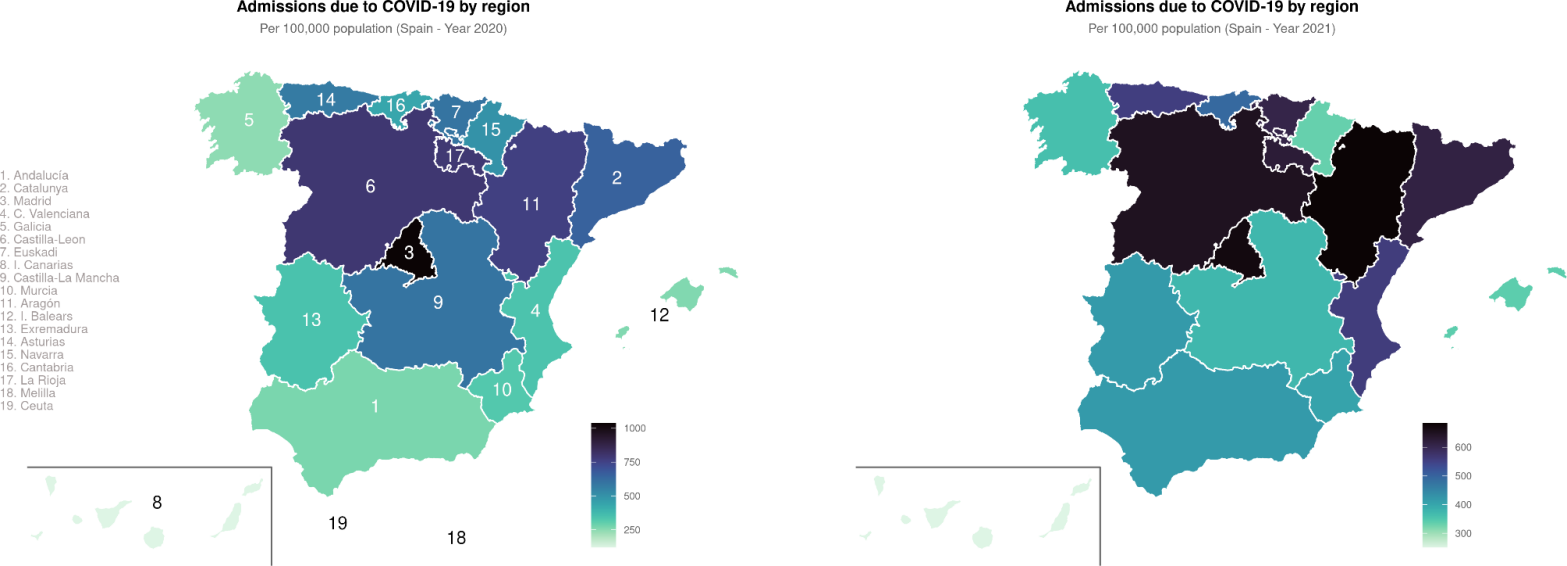
Geographic distribution of hospitalizations by region, attending to the population of each region. Data are expressed as hospitalizations per 100,000 population

The number of hospital admissions per region in Table 3 is however expressed in absolute values and might be misleading. In 2021 some regions, such as Andalucía and C. Valenciana, had more admissions than in the previous year. We thus plotted the same data, attending to the population of each region. Figure 3 shows the geographic distribution of admissions per 100,000 population. This new distribution is more homogeneous than that shown in Table 3, with less pronounced differences among regions. Nevertheless, regions such as Andalucía, Extremadura, Murcia, Galicia, and Navarra had moderately low admissions per 100,000 population compared to regions such as Madrid and Catalunya.

**Table 3:** Distribution of hospital admissions among different regions of Spain

	2020				2021			
	Admissions	Sex (male, %)	Age	Hospital stay (days)	Admissions	Sex (male, %)	Age	Hospital stay (days)
Andalucía	22,999	55.59	68 (26)	8 (9)	35,648	56.22	63 (28)	8 (9)
Aragón	10,117	53.72	73 (27)	9 (9)	9,075	55.44	66 (29)	8 (8)
Asturias	5,804	52.41	76 (24)	9 (9)	5,588	55.53	68 (26)	7 (8)
Illes Balears	3,080	58.31	63 (29)	8 (9)	4,008	56.91	60 (29)	8 (10)
Islas Canarias	2,627	57.52	66 (25)	11 (12)	5,476	56.46	60 (29)	10 (11.2)
Cantabria	2,558	53.28	71 (27)	8 (7)	2,895	53.78	63 (30)	7 (6)
Castilla-León	18,880	56.19	74 (25)	8.5 (9)	15,463	58.2	68 (28)	8 (9)
C. La Mancha	12,313	57.7	71 (24)	8 (8)	7,709	56.14	69 (27)	8 (9)
Catalunya	51,237	56.17	67 (26)	7 (8)	47,943	57.12	65 (29)	7 (9)
C. Valenciana	17,448	55.69	66 (27)	8 (8)	28,531	56.76	66 (27)	7 (8)
Extremadura	3,674	52.86	73 (24)	9 (8)	4,419	55.4	71 (27)	8 (8)
Galicia	6,620	54.32	72 (25)	9 (10)	9,775	55.81	68 (28)	8 (11)
C. Madrid	70,105	55.44	66 (28)	7 (9)	45,407	55.93	63 (28)	8 (9)
R. Murcia	5,016	55.3	60 (29)	7 (8)	6,206	56.69	62 (28)	7 (8)
Navarra	3,329	54.73	69 (27)	7 (7)	2,220	55.95	65 (30)	7 (9)
Euskadi	13,273	56.42	70 (25)	7 (8)	13,432	57.65	65 (27)	7 (8)
La Rioja	2,532	53.36	70 (27)	8 (7)	2,007	56.15	67 (28)	7 (8)
Ceuta	239	46.3	65 (22)	9 (10)	293	59.73	58 (25)	11 (11)
Melilla	325	54.15	60 (25)	8 (8)	518	50.77	59 (27)	8 (8)
**Total**	252,176	55.59	68 (28)	8 (9)	246,613	56.52	65 (28)	8 (9)

Sex is expressed as a percentage, whereas age and hospital stay are expressed as medians (interquartile ranges)

**Fig. 4:**
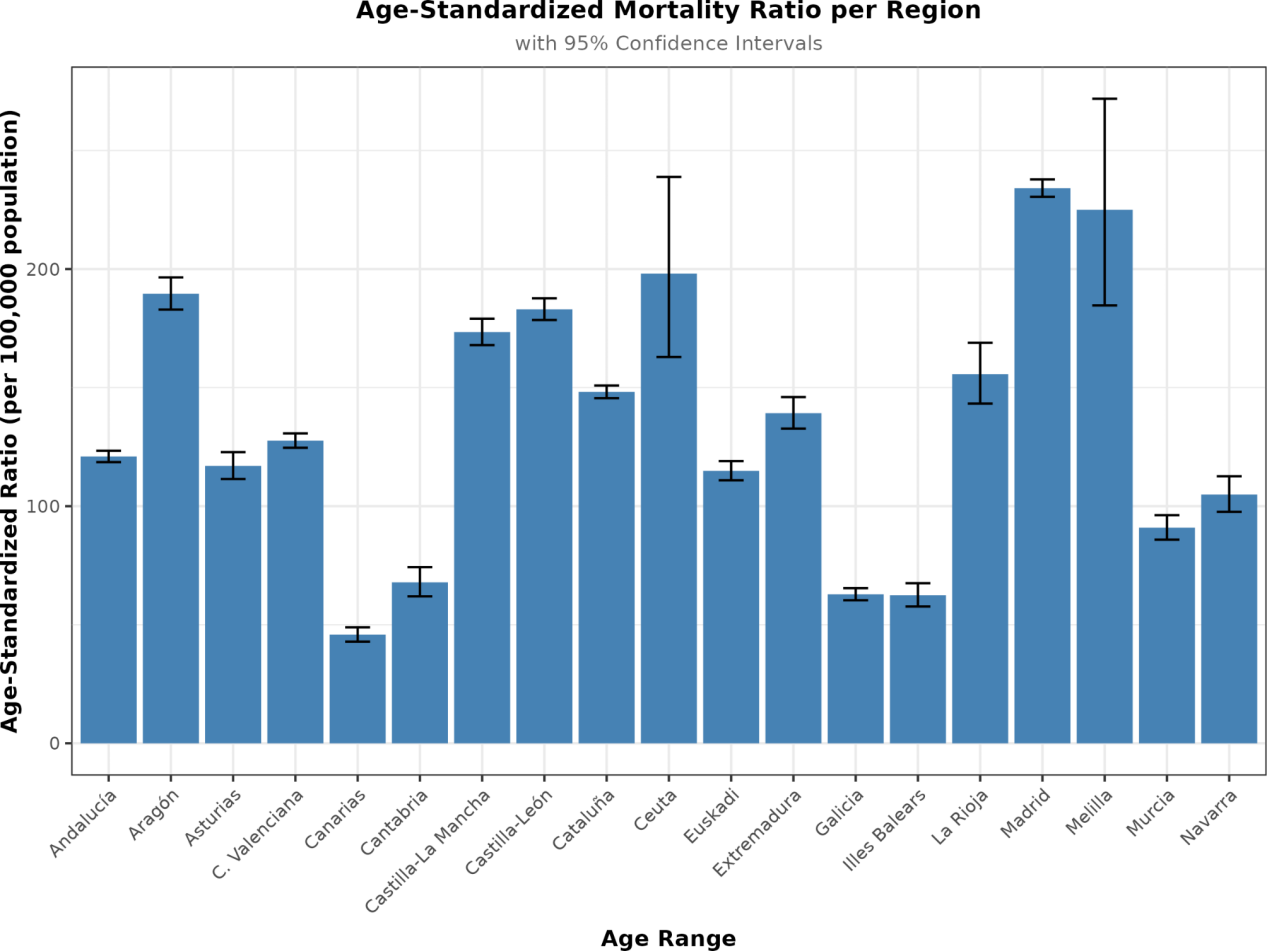
Age-standardized (adjusted) mortality rate per region, using the direct method

We calculated the crude and the age-standardized (adjusted) mortality rates per region using the direct method, showed in Table 4. Figure 4 shows the geographic distribution of age-standardized mortality.

**Table 4:** Age-adjusted (standardized) mortality rates per region, per 100,000 population

	Crude rate	Adjusted rate (95% CI)
**Andalucía**	115.9	121 (118.6–123.4)
**Aragón**	231.2	189.6 (182.9–196.5)
**Asturias**	169.4	117 (111.5–122.8)
**Illes Balears**	54.2	62.5 (57.7–67.5)
**Canarias**	41.9	45.8 (42.9–48.9)
**Cantabria**	83	67.9 (62–74.3)
**Castilla-León**	267.8	183 (178.5–187.7)
**Castilla-La Mancha**	184.8	173.4 (167.9–179.1)
**Cataluña**	153.4	148.2 (145.5–150.9)
**C. Valenciana**	134.9	127.6 (124.6–130.7)
**Extremadura**	164.1	139.2 (132.7–146)
**Galicia**	90.3	62.8 (60.3–65.4)
**Madrid**	232.5	234.1 (230.5–237.8)
**Murcia**	79.8	90.9 (85.9–96.2)
**Navarra**	116.1	104.9 (97.6–112.6)
**Euskadi**	143.7	114.9 (110.9–119)
**La Rioja**	183.5	155.7 (143.3–168.9)
**Ceuta**	138.4	198.1 (162.9–238.9)
**Melilla**	132.7	225 (184.7–271.8)

CI: Confidence interval

### Severity of COVID-19: ICU admissions and mortality

**Fig. 5:**
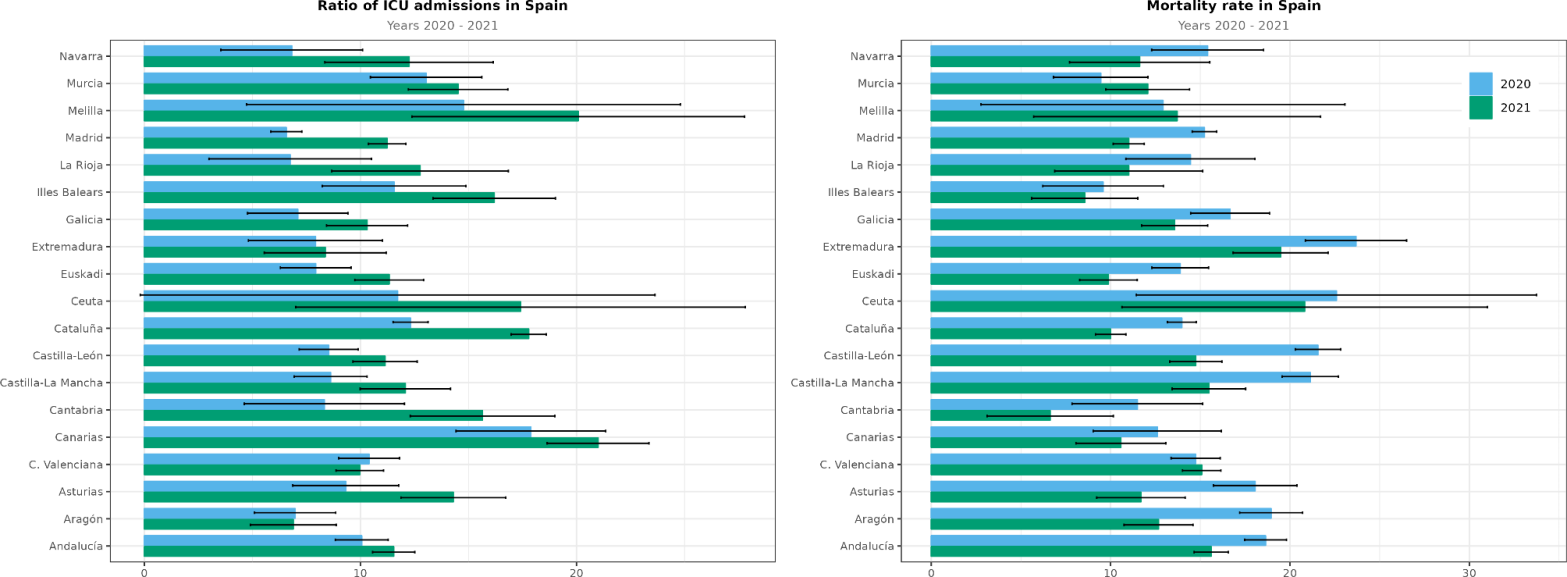
Ratios of both admissions to the intensive care unit (ICU) and deaths among hospitalized patients

We also assessed ICU admissions and mortality and their distribution by region. Table 5 shows the number of ICU admissions, the length of ICU stay, and mortality in the period studied. ICU admissions and mortality are reported as both absolute values and ratios. These ratios were calculated based on the number of hospitalized patients. Globally, there were 40,512 registered deaths in 2020 (mortality rate of 16%) and 30,925 deaths in 2021 (mortality rate of 12.5%). There were more ICU admissions in 2021 than in 2020 (Fig. 5). We also found differences among regions. In terms of mortality, Ceuta, Castilla-La Mancha, Extremadura, and Castilla-León reported the highest ratios of deaths per hospitalized patients (above 20%) in 2020. The global ratio decreased in 2021, but regions such as Ceuta and Extremadura registered the highest mortality rates in Spain. It is important to consider that when the number of events is extremely small, the calculation method for confidence intervals may result in a negative value for the lower confidence limit, as observed in the case of Ceuta. This occurrence is often attributed to the limited number of events, specifically 28 ICU admissions. Consequently, researchers should exercise caution and interpret these results with care.

**Table 5:** Distribution of ICU admissions and mortality among different regions of Spain

	2020					2021				
	ICU admissions	ICU (%)	ICU stay	Deaths	Mortality rate (%)	ICU admissions	ICU (%)	ICU stay	Deaths	Mortality rate (%)
Andalucía	2,314	10.06	10 (17)	4,287	18.64	4,115	11.54	10 (16)	5,563	15.61
Aragón	706	6.98	14 (19)	1,917	18.95	625	6.89	13 (22)	1,150	12.67
Asturias	541	9.32	10 (15)	1,048	18.06	799	14.3	14 (24)	653	11.69
Illes Balears	356	11.56	7 (19)	295	9.58	649	16.19	1 (10)	343	8.56
I. Canarias	470	17.89	11 (19)	331	12.6	1,150	21	11 (19)	579	10.57
Cantabria	213	8.33	9 (12)	294	11.49	453	15.65	9 (13)	192	6.63
Castilla-León	1,611	8.53	11 (16)	4,070	21.56	1,722	11.14	13 (24)	2,281	14.75
C. La Mancha	1,061	8.62	11 (19)	2,602	21.13	931	12.08	12 (22)	1,193	15.48
Catalunya	6,313	12.32	8 (14)	7,156	13.97	8,527	17.79	8 (12)	4,795	10
C. Valenciana	1,815	10.4	9 (14)	2,572	14.74	2,845	9.97	11 (17)	4,303	15.08
Extremadura	291	7.92	13 (16)	870	23.68	370	8.37	13.5 (16)	861	19.48
Galicia	470	7.1	9 (13)	1,103	16.66	1,008	10.31	10 (15)	1,326	13.57
C. Madrid	4,608	6.57	11 (17)	10,676	15.23	5,105	11.24	22 (31)	4,999	11.01
R. Murcia	654	13.04	9 (13)	474	9.45	901	14.52	8 (15)	749	12.07
Navarra	227	6.82	17 (25)	513	15.41	272	12.25	10 (18)	258	11.62
Euskadi	1,052	7.93	11 (17)	1,842	13.88	1,522	11.33	11 (18)	1,327	9.88
La Rioja	171	6.75	11 (13)	366	14.45	256	12.76	15 (21)	221	11.01
Ceuta	28	11.72	15 (25)	54	22.59	51	17.41	13 (19)	61	20.82
Melilla	48	14.77	7.5 (9)	42	12.92	104	20.08	6 (11)	71	13.71
**Total**	22,949	9.1	10 (16)	40,512	16.06	31,405	12.73	11 (19)	30,925	12.54

ICU: intensive care unit. ICU and mortality rates are expressed as percentages, whereas ICU stay is expressed as a median (interquartile range)

## Discussion

The main objective of our nationwide study was to analyze two available years of data on hospitalizations, ICU admissions, and mortality due to COVID-19 provided by the Spanish National Health System. The COVID-19 outbreak overwhelmed the capacity of hospitals, and our aim was to assess its impact on the Spanish population. To the best of our knowledge, this is the first Spanish nationwide research on this topic.

Spain was one of the epicenters of the pandemic in Europe, with high rates of both hospitalizations and mortality, especially among the elderly. Specifically, Madrid had the highest rate of confirmed cases and hospitalizations [14, 18]. We found that in the first 2 years of the pandemic, almost 500,000 patients in Spain needed hospitalization, and more than 70,000 died. The COVID-19 pandemic overwhelmed hospital wards and ICUs and subsequently the capacity of the health care system.

The burden in terms of hospitalization, ICU admissions, death, and health care capacity varied by region. We found great variation in hospitalizations and deaths across regions, which highlights the different impacts on each region, even if hospital admissions are adjusted by population. The reasons for such differences are unclear, and a comparison of clinical outcomes and economic factors or sociodemographics by region is beyond the scope of this research; however, we can raise a hypothesis of economic disparities among regions [19]. Researchers studied geographic variability in the relationships between mortality rate and social, demographic, and health care factors in Spain during the first wave of the pandemic [20]. The unemployment rate, population density, and population size were the main underlying factors related to geographic variation in risk of death. The unemployment rate is an indicator of socioeconomic status, and regions with high unemployment invest less in education and health care. Also, population density is associated with social distancing, which emphasizes the importance of restrictive measures. Although the researchers encouraged further research, their data provide critical information for mitigating the impact of COVID-19 and future pandemics. This raises the question of how different regions fare during public health emergencies and can identify areas that can be improved for future outbreaks.

Several researchers have reported that older age is associated with hospitalization [13, 21]. Likewise, we found that 77.4% of hospitalizations were among individuals older than 50, 42.7% were among those older than 70, and 23.4% of all hospital admissions were among patients older than 80. Moreover, for each additional decade of life, the risk increased by 71.5%. Although this was not a correlational study, we found that age was probably the major factor associated with hospitalization due to COVID-19.

Regarding the distribution of waves over time, we can hypothesize only about the association—not causation—between certain events and peaks of hospitalizations in Spain. The first wave was associated with the initial outbreak and was restrained by strict public health measures, such as confinement and lockdown. The second wave began in the summer of 2020, when those restrictions ended and social distancing measures were relaxed. This wave reached a peak in the autumn of 2020, probably because of the return to work and school. The third peak began in December 2020, probably as a result of holiday events and Christmas gatherings, and continued until January 2021. The fourth wave showed a rapid fall in hospitalizations, probably because of vaccination, and its peak might have been associated with the Easter holidays. The fifth and the sixth waves (September 2021 and December 2021, respectively) were similar to the fourth wave in terms of hospitalizations, and they showed the beneficial effect of vaccination. Regardless of vaccination, it seems that waves and peaks were related to social events: holidays, gatherings, and the relaxation of public health measures such as social distancing.

In the summer of 2020 new drugs began to be used to treat COVID-19 in hospitalized patients, such as remdesivir; corticosteroids [22, 23]; and immunomodulatory drugs such anakinra, tozilizumab, and baricitinib [24–29]. The use of these new drugs can explain the lower mortality in 2021 [30].

Other Spanish studies have also identified several waves, characterized as peaks in the incidence of confirmed cases of COVID-19. It is worth noting an epidemiological study by Red Nacional de Vigilancia Epidemiologica (RENAVE) in which data from Spain were reported [18]. In this study, confirmed cases, hospitalizations, and deaths were analyzed through May 10, 2022. The researchers split the pandemic into periods and established a turning point for each wave based on the 14-day cumulative incidence. Waves in that research are in line with the ones in our study (i.e., their peaks occurred simultaneously with the waves of hospitalizations and deaths identified in our research). We found that after the first outbreak of COVID-19, hospitalizations increased over the first three waves of the pandemic. After that, hospitalizations underwent a significant decline. Early mass vaccination probably delayed and alleviated the fourth wave, which finally occurred in the spring of 2021.

The most important public health measure during the pandemic was vaccination. Vaccination had begun in the European Union by January 2021. Its effects, in terms of admission and mortality, were not evident until April 2021. Beginning in December 2020, vaccines were administered to the entire Spanish population, and they proved significant protection against severe COVID-19. The first peaks before vaccination occurred in March 2020, November 2020, and January 2021. Beginning in the spring of 2021, the use of vaccines resulted in a decrease in hospitalizations, probably because their protection was based on achieving a mild clinical presentation of COVID-19. They had a great impact in terms of hospital admissions and mortality. The effect of vaccination on the fourth wave was studied elsewhere [21]. Moreover, we demonstrated that this impact was steady in the fifth and sixth waves. It is of interest to mention a study by Barandalla et al. [21], who developed simulated curves of hospitalizations in the absence of vaccines and then compared those curves with the real incidence. By showing the decrease in incidence, they demonstrated the beneficial impact of the vaccination rollout on hospitalizations.

Several noteworthy caveats should be mentioned. For one thing, several variants of the SARS-CoV-2 virus were identified throughout the pandemic. The changes in SARS-CoV-2 variants can also explain the beginning of certain waves. In the summer of 2020 the alpha variant (B.1.1.7) was described [12], and this variant spread rapidly in several European countries. It had greater transmissibility, virulence, and lethality than previous variants and could have been responsible for the second and third waves. Alpha was replaced by delta (B.1.617.2) in Spain in the summer of 2021 and could have been one of the causes of the peak of the fifth wave. Nevertheless, there is no evidence that delta or other new variants had a negative impact on hospitalizations after the summer of 2021, as clinical presentations were often mild [31] and vaccination against these two variants was effective [32, 33].

Herd immunity is another caveat. An interesting Spanish study (Estudio Nacional de sero-Epidemiología de la Infección por SARS-CoV-2 en España, or ENE-COVID) analyzed seroprevalence after the first wave [34]. Only 5% of the entire Spanish population had antibodies against SARS-CoV-2, but measurements varied across different regions. Madrid, Castilla-La Mancha, and Castilla-León (central regions) had seroprevalence above 10% compared to approximately 1% in regions such as Galicia and C. Valenciana (peripheral regions along the coast). Herd immunity was not achieved in Spain after the first wave, which clearly rendered vaccination a key factor in achieving protection against SARS-CoV-2.

ENE-COVID not only demonstrated that there were several epicenters during the pandemic, such as Madrid, but also highlighted the proportion of symptomatic individuals who were not diagnosed by a proper polymerase chain reaction test. Concerning testing policy, a recent publication by Zhang et al. [35] demonstrated that mass testing was associated with a 25% decrease in hospitalizations due to COVID-19. The city of Liverpool (United Kingdom) was selected for a pilot study. The intervention involved testing asymptomatic individuals to identify infected people to protect vulnerable individuals, quarantine contacts, and ultimately improve public health. This intervention reduced COVID-19-related admissions because promoting effective isolation of confirmed individuals and their contacts resulted in reduced onward transmission. The study estimated a 32% reduction in admissions compared to the expected admissions with no intervention. ENE-COVID demonstrated that underdiagnosis was common during the first weeks of the pandemic. Testing policy and their impact on the COVID-19 pandemic in Spain before vaccines were available were also studied by several researchers [30, 36]. The first three waves were analyzed, and several differences were found between the first wave (with its more restrictive testing policy) and the second and third ones (with their less restrictive policies). These researchers demonstrated not only an increase in the number of confirmed cases in the general population but also decreases in the number of severe cases requiring treatment in the ICU and in mortality rates during the second and third waves compared to the first wave. In the summer of 2020 a less restrictive testing policy began to be effectively implemented in Spain, ensuring the detection of cases with mild symptoms and even asymptomatic individuals. These interventions contributed to addressing the outbreaks, as pointed out by another study that demonstrated that underreporting cases of COVID-19 can lead to poor outcomes [37]. The more cases are reported, the more able experts are to trace confirmed cases and to maintain quarantine. Thus, nonpharmaceutical interventions such as restrictive testing policies, confinement, masks, restricted mobility, and social distancing can also have a great impact on COVID-19.

### Limitations: the reliability of administrative data

The main limitation of our study was our use of an administrative database. Although electronic health records help researchers collect data and make them available for research, some clinical data are not readily available. Information about vaccination or drugs were not recorded, so we could not assess the effect of vaccination or certain treatments. This issue is inherent to the MBDS-H because of the nature of its coding process. Also, we only had access to data from 2020 to 2021, because data from 2022 had not yet been processed. This limitation causes a delay in reporting epidemiological data such as ours. Another limitation our of study has to do with the reliability of the data. Although it is based on discharge reports, the MBDS-H was primarily designed as an administrative data set focused on economical management rather than clinical relevance [38–40]. Diagnoses are recorded and coded, but data regarding diagnostic procedures, treatments, and outcomes are not registered. Moreover, the validity of the collected data relies on the accuracy of the medical discharge reports and the recording of the variables. It is worth noting that diagnoses may have been provisional or redundant, and some of them may have been excluded if administrative personnel could not find an appropriate code in the ICD-10-CM. The administrative personnel may not have had any medical training. On the whole, we can state that the reliability of such databases may be not guaranteed [41]. However, although a certain amount of misclassification is expected, reliability can be improved with strategies such as examining secondary diagnoses [42]. In contrast, some have argued that the MBDS-H provides sufficiently valid information and can be a useful tool in epidemiological and clinical studies [43, 44].

## Conclusions

Here we offered a picture of SARS-CoV-2-related hospitalizations in Spain in the first 2 years of the pandemic. Our aim was to describe trends and the distribution of the pandemic in Spain and to determine the impact of the pandemic on the Spanish health care system and population. We identified at least six waves over the 2 years. We also found differences between the years in terms of the number of hospitalizations, ICU admissions, and deaths. We proposed explanations for the rise and fall in hospitalizations, such as public health measures or social distancing, but we are aware that certain factors may have played an important role in the waxing and waning of each wave, such as vaccination rates or the predominant variant of the virus. Vaccination was probably the most important public health intervention to mitigate the pandemic, as it gave individuals protection against severe COVID-19. The distributions of both hospital admissions and mortality showed great differences across regions of Spain, probably because of underlying socioeconomic factors.

## Data Availability

According to the terms of a contract signed with the Spanish Ministry of Health, which provided the data, the authors cannot provide the data set to any other researcher. Furthermore, the data must be destroyed at the conclusion of the research. Data can be obtained at https://www.sanidad.gob.es/estadEstudios/portada/home.htm.
